# VEGF and eNOS Expression in Umbilical Cord from Pregnancy Complicated by Hypertensive Disorder with Different Severity

**DOI:** 10.1155/2014/982159

**Published:** 2014-05-14

**Authors:** K. Bhavina, J. Radhika, S. Sundara Pandian

**Affiliations:** SRM Medical College Hospital & Research Center, Kattankulathur, Kanchipuram, Tamil Nadu 603203, India

## Abstract

*Background*. Reduced blood flow in hypertensive pregnancy may influence the production vasoconstrictors; subsequently the vessel remains in highly contracted state. NO is a vasodilator; VEGF influences its synthesis by regulating eNOS production. Aim of our study was to evaluate the expression of VEGF and eNOS in different severity of hypertensive pregnancy. *Methods*. Study was conducted in 4 groups with 40 members: group 1—control, group 2—gestational hypertension, group 3—mild preeclampsia, and group 4—severe preeclampsia. Fetal end of umbilical cord was taken and follows IHC staining protocol for VEGF and eNOS antibody. Staining intensity were measured by semiquantitative scoring method. Mann Whitney *U* test was used to compare each group. *Results*. Decreased expression of both VEGF and eNOS was found in hypertensive condition than in normal condition. Among hypertensive group, severe preeclamptic group showed more intensity in staining than gestational hypertension and mild preeclampsia. *Conclusion*. Reduction of VEGF and eNOS in gestational hypertension may lead to hypoperfusion and subsequent hypoxia of fetus in hypertensive pregnancy. The developed hypoxic state may upregulate the synthesis of VEGF and thereby eNOS. Increased expression of VEGF and eNOS in severe group may be a compensatory mechanism to dilate the blood vessels and to improve blood flow of fetus.

## 1. Introduction

Hypertension is the common pathology related to pregnancy and occurs in 4-5% and it is the major cause of maternal and fetal mortality and morbidity [1]. Normally, during pregnancy, spiral arteries of uterus undergo a remodeling by the invasion of cytotrophoblast in their walls and thereby reduce a resistance in blood flow to intervillous spaces [2, 3]. Inadequate invasion of trophoblast results in placental ischemia and development of hypertension [4–6]. Subsequently it leads to fetal hypoxia and it results in low birth weight and these babies are more prone to acquire hypertension in later life [7].

Umbilical cord does not have any nerve supply, so the structure of umbilical vessels was purely depending on rate of blood flow through it [8]. Reduced blood flow in hypertensive pregnancy may influence the production of vasoconstrictors and subsequently the vessel remains in highly contracted state [9]. The action of vasoconstrictors may be attenuated by vasodilators [10]. The chief vasodilator is nitric oxide. The nitric oxide is synthesized from L-arginine; it is catalysed by the enzyme eNOS [11]. Studies have demonstrated that VEGF is a potent endothelial cell mitogen, which can stimulate the proliferation of endothelial cells, microvascular permeation, vasodilation, and angiogenesis [12, 13]. Vasodilation is possible by the induction of nitric oxide by regulating eNOS expression [14, 15]. Shen et al. observed an increased activity and expression of eNOS in endothelial cell culture with prolonged exposure in VEGF [16]. In this way VEGF can improve the endothelial function and it was regulated by hypoxia [17].

Studies have proved the occurrence of hypoxia in hypertensive pregnancy and the condition became worse along with its severity. Different opinions have been observed in the expression of VEGF and eNOS in umbilical cord of preeclampsia. The aim of the present study is to evaluate the expression of VEGF and eNOS in umbilical cord from pregnancies complicated by hypertensive disorder with different clinical severity and to attempt to clarify the disparity.

## 2. Materials and Methods

### 2.1. Patients

Four groups were considered for our study. Each group contains 40 members. Group 1 was control group, women with uncomplicated pregnancy. Group 2 was gestational hypertensive group, comprising women with blood pressure ≥140/90 after 20 weeks of gestation but no proteinuria. Group 3 was women with mild preeclampsia, who had sustained blood pressure ≥140/90 and proteinuria 1+ in dip stick but they had normal BP and proteinuria before 20 weeks. Group 4 was severe preeclamptic women with blood pressure ≥160/110 and proteinuria 3+ in dip stick, in every 6 hours of evaluation. Women with chronic hypertension, renal failure, cardiac problems, and multiple pregnancies were excluded.

Institutional ethical committee has approved the study. Placentas with umbilical cord of these groups were used for the present study and these were collected from SRM Medical College Hospital and Government Hospital for Women and Children, Egmore.

### 2.2. Slide Preparation

1 cm length of the cord was taken from its fetal end, that is, 2 cm away from cut end after clamping. The segments were fixed in 10% of formalin and followed by the routine steps for tissue processing and embedded in paraffin wax. Four micron thick sections on polylysine coated slides were used for immunohistochemical procedures. Before starting the IHC protocol, sections were dewaxed and rehydrated. Antigen retrieval was performed by using microwave oven for 15 minutes. Then the slides were treated with 3% hydrogen peroxide block to remove endogenous peroxidase activity. After washing with PBS, enough drops of primary antibody for VEGF (Thermo Scientific, rabbit polyclonal antibody) and eNOS (Thermo Scientific, rabbit polyclonal antibody) were separately added and incubated for 1 hour. Then secondary antibody was added. Reaction was visualized with DAB. The slides were counterstained with haematoxylin.

### 2.3. Evaluation

Semiquantitative scoring method was used to evaluate the expression of VEGF and eNOS in tissues. Based on the intensity of the staining, the antibodies present in the cells were scored as 0 (no staining), 1 (weak staining), 2 (moderate staining), and 3 (strong staining). Expression of antibodies scored separately in amniotic epithelium, Wharton's jelly, endothelium, and smooth muscle cells of artery and vein. All immunostained sections were reviewed by a histologist, who was blind to the purpose of study. She calculated the* H* score by multiplying the intensity of staining with percentage of cells stained. Calculated* H* score values were between 0 and 300. Based on these values again the staining intensity divides into 0–3+.* H* score value up to 100 is 1+, between 101 and 200 is 2+, and from 201 to 300 is 3+. Randomly selected fields were used for scoring.

### 2.4. Statistics

Mann whitney* U* test was used to compare the staining intensity between every two groups. *P* value ≤0.05 was considered statistically significant. Statistical analysis was done by using SPSS version 7.5.

## 3. Results

Clinical details of study groups are given in Table 1. Baby weight, placental weight, gestational age, and Apgar score were decreased in hypertensive condition when comparing with normal condition. Among the PIH group, the lowest values were observed in severe preeclampsia. Placental abruption was also noted only in preeclampsia, that is, one case in mild preeclampsia and three cases in severe. In severe preeclamptic group, intrauterine death was reported in two cases.

**Figure 1:**
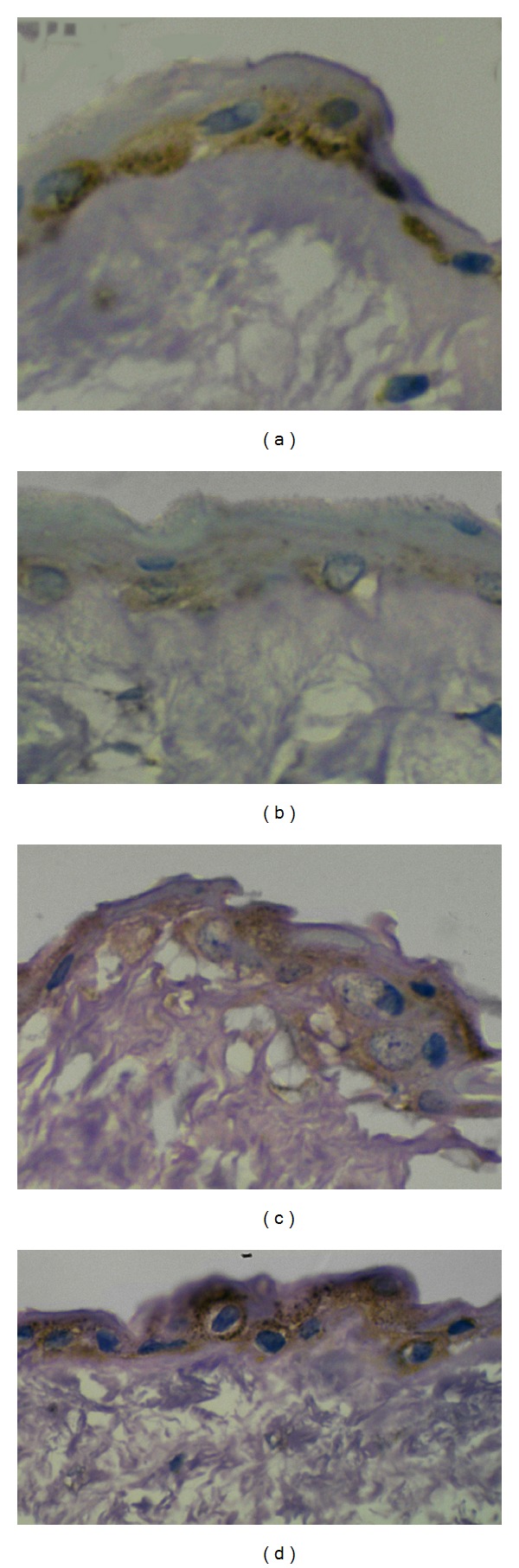
Expression of VEGF in amniotic epithelium: (a) control group; (b) gestational hypertension; (c) mild preeclampsia; (d) severe preeclampsia.

Immunoreactivity of VEGF was given in Table 2. Positive immunoreactivity for VEGF was found in cells of amniotic epithelium, Wharton's jelly, epithelium, and muscle tissue of umbilical artery and vein. When comparing with normal, intensity of staining was shown as a significant reduction in hypertensive group. Among the hypertensive group, a high intensity of staining in all tissues of severe preeclampsia and a least staining in gestational hypertension was observed. Figures 1(a)–1(d) are showing the differences in intensity of staining in amniotic epithelial cells among 4 groups. VEGF expression in amniotic epithelium of severe preeclampsia was almost the same as normal. No significant difference in the expression of VEGF was observed between gestational hypertension and mild preeclampsia, except in amniotic epithelium.

**Figure 2:**
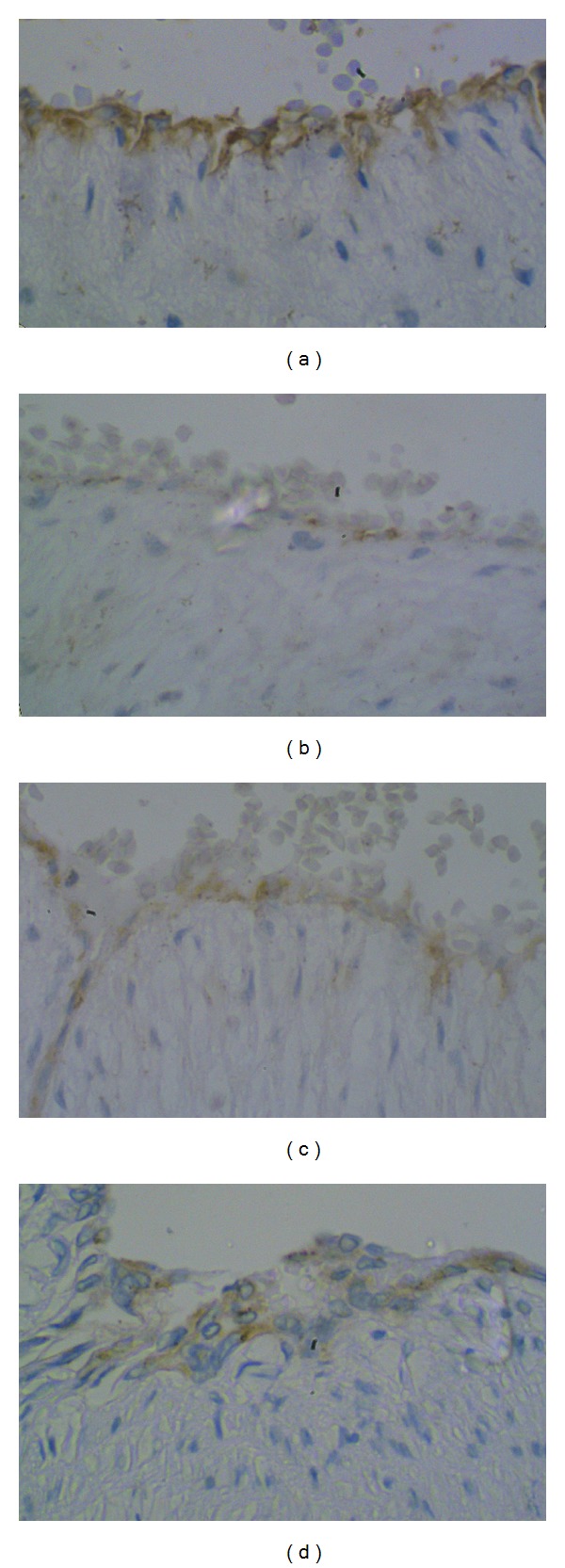
eNOS expression in endothelium of umbilical artery: (a) control group; (b) gestational hypertension; (c) mild preeclampsia; (d) severe preeclampsia.

The expression of eNOS has been shown in Table 3. eNOS expression is present only in endothelium of artery and vein. Its expression was also significantly less in hypertensive group than normal group. In the hypertensive group, here also a progressive increase in staining was seen along with its severity, Figures 2(a)–2(d) showing the differences in expression of eNOS in artery endothelium of 4 groups. Severe preeclampsia with intrauterine fetal death cases did not show much increase in the expression of these factors.

## 4. Discussion

This is the first study to investigate the expression of VEGF and eNOS in umbilical cord components from pregnancy complicated with different severity of hypertension. Many studies were conducted on preeclampsia with regard to control group. Site of location of VEGF and eNOS was the same as previous studies [18, 19]. Acute reduction of VEGF may induce hypertension [20]. Anti-VEGF drugs used in cancer treatment might cause the development of hypertension [21].

Similar to the other previous study results, we also observed a significant reduction in intensity of staining in hypertensive group, when comparing it with control [22, 23]. But among the hypertensive group, the least staining intensity of VEGF and eNOS was noticed in gestational hypertension. That means, when the hypertensive state progresses from gestational hypertension to severe preeclampsia these angiogenic factors become increased. In contrary to that, some investigators found an increased expression of VEGF in preeclampsia than in normal [24].

As a result of high resistant placenta, blood flow through umbilical vessels was decreased in hypertensive disorder [6]. To adapt the low blood flow, the umbilical vessels were altered its structure by increasing its thickness [9]. It may be influenced by reduced production of nitric oxide. Nitric oxide has a protective role by inhibiting the proliferation of smooth muscle cells in vessel wall [25]. This study also observed a further increase in expression of VEGF and eNOS in severe preeclampsia. Progressive condition of hypertension is associated with hypoperfusion that may lead to hypoxia. VEGF production was upregulated by hypoxia that might be the reason for the observed increased expression of the same in preeclampsia when it progresses from gestational hypertension to severe.

VEGF induces the synthesis of nitric oxide, which is a potential vasodilator. Prolonged exposure of endothelium to VEGF in culture leads to high synthesis of nitric oxide by the influence of eNOS [16]. The expression of eNOS in endothelial cells was found to be decreased in hypertensive group than normal group, it might be the effect of reduction of VEGF. Nitric oxide could attenuate the action of vasoconstrictors [10]. In hypertension, due to the uncontrolled action of vasoconstrictors, the umbilical vessels remain in constricted state and develop more hypoxic state [9]. As a compensatory mechanism, VEGF was upregulated by this hypoxia, which in turn influences the production of eNOS and thereby nitric oxide to facilitate the blood flow to fetus.

## 5. Conclusion

The reduction of VEGF and eNOS in umbilical cord might be the cause of development of hypoperfusion and subsequent initial hypoxia of fetus in hypertensive pregnancy. Developed hypoxia may upregulate the production of VEGF in the progress of pathology. VEGF stimulates the production of eNOS and thereby nitric oxide. With regard to the severity of pathology an increased production of VEGF and eNOS might be the compensatory mechanism to dilate and to improve the blood supply to fetus.

## Figures and Tables

**Table 1:** Comparison of clinical data.

	Normal	GH	PE mild	PE severe
Systolic pressure in mm Hg	≤120	≥130	≥140	≥160
Diastolic pressure in mm Hg	≤80	≥90	≥90	≥110
Proteinuria	Nil	1+	2+	≥3+
Edema	Occasionally	Occasionally	Present	Present
Birth weight in gm	2923.5 ± 326.43	2702.5 ± 387.62	2410 ± 529.3	2172.5 ± 374.33
Placental weight in gm	455.63 ± 47.87	383.75 ± 71.29	327.5 ± 83.17	290 ± 443.41
Gestational age in weeks	38.15 ± 1.48	37.55 ± 1.62	36.55 ± 2.73	35.45 ± 1.84
Apgar score—1 minute	7.88 ± 0.33	6.9 ± 0.3	6.65 ± 0.66	6.2 ± 2.11
Apgar score—5 minutes	8.88 ± 0.33	7.9 ± 0.3	7.65 ± 0.8	7.2 ± 2.43
Intrauterine death	Nil	Nil	Nil	2
Placental abruption	Nil	Nil	1	3

**Table 2:** Localization and immunostaining intensity of VEGF expression in umbilical cord tissue.

	Control—*N* (%)	GH—*N* (%)	PE mild—*N* (%)	PE severe—*N* (%)
Amniotic epithelium				
1+	0	65 (26)*	10 (4)^∗#^	0^#$^
2+	45 (18)	30 (12)	58 (23)	45 (18)
3+	55 (22)	5 (2)	32 (13)	55 (22)
Wharton's jelly				
0	0	5 (2)*	0*	0
1+	32 (13)	85 (34)	75 (30)	55 (22)^∗#$^
2+	38 (15)	10 (4)	25 (10)	28 (11)
3+	30 (12)	0	0	17 (7)

Artery endothelium				
0	0	43 (17)*	40 (16)*	0^∗#$^
1+	68 (27)	57 (23)	60 (24)	100 (40)
2+	30 (12)	0	0	0
3+	2 (1)	0	0	0
Artery muscle cells				
1+	65 (26)	100 (40)*	100 (40)*	83 (33)^∗#$^
2+	35 (14)	0	0	17 (7)
Vein endothelium				
0	0	50 (20)*	30 (12)*	0^∗#$^
1+	85 (34)	50 (20)	70 (28)	100 (40)
2+	15 (6)	0	0	0
Vein muscle cells				
0	0	30 (12)*	18 (7)*	0^∗#$^
1+	80 (32)	70 (28)	82 (33)	80 (32)
2+	20 (8)	0	0	20 (8)

GH: gestational hypertension, PE: preeclampsia, *N*: number of specimens, %: percentage.

*Significant = *P* < 0.05 control versus GH, PE mild, PE severe. ^#^Significant = *P* < 0.05 GH versus PE mild, PE severe. ^$^Significant = *P* < 0.05 PE mild versus severe.

**Table 3:** eNOS expression in different cell components of umbilical cord.

	Control	GH	PE mild	PE severe
Artery endothelium				
0	0	25 (10)*	23 (9)^∗#$^	0^∗#$^
1+	7 (3)	58 (23)	27 (11)	5 (2)
2+	15 (6)	17 (7)	50 (20)	50 (20)
3+	78 (31)	0	0	45 (18)
Vein endothelium				
1+	10 (4)	100 (40)*	38 (15)^∗#$^	15 (6)^#$^
2+	12 (5)	0	22 (9)	28 (11)
3+	78 (31)	0	40 (16)	57 (23)

GH: gestational hypertension, PE: preeclampsia, *N*: number, %: percentage.

*Significant = *P* < 0.05 control versus GH, PE mild, PE severe. ^#^Significant = *P* < 0.05 GH versus PE mild, PE severe. ^$^Significant = *P* < 0.05 PE mild versus severe.
